# The Hospitals' Harvest Time

**Published:** 1916-09-09

**Authors:** 


### The Hospital

The Workers' Newspaper of Administrative Medicine and Institutional Life, Administration, National Insurance and Health.

No. 1579, Vol. LX. SATURDAY, SEPTEMBER 9, 1916. PRICE ONE PENNY.

### THE HOSPITALS' HARVEST TIME.

How many hospital workers who have to raise large sums every year from voluntary contributions, by the keeping of accurate records, and the continuous study of the yield from voluntary gifts, can convincingly show which season of the year yields the fullest returns to the hospital exchequer, and so may be regarded as the hospitals' harvest time? At a recent meeting of the Metropolitan Hospital Sunday Fund the Rev. W. Hardy Harwood carried a resolution instructing the General Purposes Committee to consider the practicability of establishing a fixed date for Hospital Sunday, preferably the second Sunday in June, and to report to the Council before the annual meeting of constituents in December. The Rev. Hardy Harwood accepted an amendment by Viscount Knutsford to add the words, " or some earlier date in the year," *i.e.* than June. In this it must be said that, consciously or unconsciously, the Rev. Hardy Harwood has raised the question which of fifty-two Sundays is, in fact, the yearly harvest day for the hospitals, so far as concerns the yield in pounds sterling which can be garnered through the churches and places of worship of all denominations on Hospital Sunday for the support of our voluntary hospitals. For forty years, subject to the varying date of Trinity Sunday, the collections have been fixed to take place early in June each year. We believe that this time was chosen originally because it was thought that the day held a central position in the period during which the majority of the wealthier and well-to-do members of the community flocked to the Metropolis in order to enjoy the season and take part in its gaieties, festivities, and amusements. Further, the day was fixed after careful inquiry so as to enable the appeal to reach a large number of wealthy visitors from the United States and other countries, who often are to be found in London in June.

Lord Knutsford, in support of his amendment, held the view that an earlier period of the year for Hospital Sunday would prove more productive, seeing that a more natural and direct appeal could be made in cold weather, and that the attendance in churches was better in winter than in summer. A life's experience leads the writer to doubt very much the soundness of the views expressed, seeing that all evidence points to the yield in charitable gifts being much more largely dependent upon the possession of a good balance at the bankers at a particular period than the uninitiated have any idea of. There can be no question that the June collections have produced in the past considerable sums derived from the presence of casual visitors, especially of those who reside habitually in the country, and have to meet the claims of the hospitals situated in the districts where they live. The same remark applies to the presence of wealthy and well-to-do strangers who are not to be found in any numbers in the Metropolis in the winter.

It will thus be seen that a heavy responsibility has been thrown upon the General Purposes Committee of the Sunday Fund by the Council, and we hope that Sir Ernest Tritton, Bart., the Deputy Chairman of the Fund, will set inquiries on foot, have the discussions looked up which took place when the collections were fixed in June, and prepare as many facts as possible from every aspect that in any way bears upon the yield of charitable gifts to institutions at particular seasons of each year. We are strongly of opinion that if there is to be any change the month chosen should be November, and that the first or second Sunday in that month should be Hospital Sunday as a fixed and unalterable date.

We are in favour of Mr. Harwood's contention that the fixed date would be likely to increase the yield, as it is usual for the churches to make their arrangements a year in advance. A fixed date should further render it impossible for any lukewarm head of a congregation to change the day on which he had collections for the hospitals, a practice which has cost the Fund many thousands of pounds in the past, and has caused many dissatisfied members of congregations so treated to attend other places of worship, and even, it is said, to leave a congregation. Hospital Sunday is the day on which all religious denominations have an opportunity of becoming united on a common platform on behalf of the sick and suffering, who are entitled to receive the support of every Christian man and woman everywhere always. This fact, widely published and eloquently driven home from every pulpit in the Metropolis, not only on Hospital Sunday, but on, at least, the Sunday preceding this day, should result, as it did in Birmingham, in reaching the hearts and consciences of the overwhelming majority of the people of all denominations. So universal did the practice become in Birmingham that the churches were more crowded on Hospital Sunday than on any other day of the year, the people made it a practice to save up and take their gifts to the churches, and the whole family, including those members who were not habitual attenders at a place of worship, were drawn together as a sacred duty to attend some place of worship as a privilege on that day.

Lord Kitchener, in one of his last statements, is said to have declared that the great need of the nation was more religion among the people. We make bold to think that in hospital matters what Hospital Sunday needs is more religion in the pulpits on that day as evidence of the preacher's recognition of his own and his people's spirituality, and its reality and living force in their lives and work. Then there would be no church where those responsible would ever again make the hospital collections a matter of little importance, for all would feel it to be a privilege religiously to hold the Hospital Sunday collections on the appointed day. The idea that a large collection on Hospital Sunday means less funds from the congregations for church purposes is as ill-founded as it is discreditable. Those clergy and ministers who have the largest Hospital Sunday collections succeed in raising the largest sums for other purposes in the course of each year. The fact is the getting of money means the wider spreading of the net, and it is necessary for religious leaders, as for all responsible for raising voluntary gifts, to extend their efforts far and wide, because the nearer the preachers get to the periphery, the steadier and larger will be the flow of money through church collections each year.

